# Single/joint effects of pyrene and heavy metals in contaminated soils on the growth and physiological response of maize (*Zea mays* L.)

**DOI:** 10.3389/fpls.2024.1505670

**Published:** 2024-12-02

**Authors:** Yuhui Wang, Muhammad A. Imran, Juanjuan Zhao, Muhammad Sultan, Manjie Li

**Affiliations:** ^1^ College of Environmental Science and Engineering, Donghua University, Shanghai, China; ^2^ Institute for Ocean Engineering, Shenzhen International Graduate School, Tsinghua University, Shenzhen, China; ^3^ Shenzhen Key Laboratory of Advanced Technology for Marine Ecology, Shenzhen International Graduate School, Tsinghua University, Shenzhen, China; ^4^ Department of Agricultural Engineering, Bahauddin Zakariya University, Multan, Pakistan

**Keywords:** contaminated soil, maize, pyrene, Cu and Cd, joint stress, growth inhibition

## Abstract

The widespread presence of polycyclic aromatic hydrocarbons (PAHs) and toxic heavy metals in soils is having harmful effects on food crops and the environment. However, the defense mechanisms and capacity of plants to counteract these substances have not been comprehensively explored, necessitating a systematic categorization of their inhibitory effects. Accordingly, an experimental investigation was conducted to examine the growth and physiological response of maize (*Zea mays* L.) to different concentrations and combinations of pyrene, copper (Cu), and cadmium (Cd), with an indicator developed to assess the joint stress. The results showed that 57-day culture with contaminations significantly inhibited the plant biomass via causing root cell necrosis, inducing lipid peroxidation, and damaging photosynthesis. Cd (50-100 mg/kg) induced stronger inhibition than Cu (800-1000 mg/kg) under both single and joint stress, and their co-existence further aggravated the adverse effects and generated synergetic inhibition. Although the presence of pyrene at a low concentration (5-50 mg/kg) can somewhat diminish the metal stress, the elevated pollutant concentrations (400-750 mg/kg pyrene, 50-100 mg/kg Cd, and 800-1000 mg/kg Cu) switched the antagonistic effect to additive inhibition on maize growth. A satisfactory tolerance of a low-level pyrene and/or metal stress was determined, associated with a relative stability of chlorophyll-a (Chl-a) content and antioxidant enzymes activity. Nevertheless, the photosynthesis and antioxidant system were significantly damaged with increasing contaminant concentrations, resulting in chlorosis and biomass reduction. These findings could provide valuable knowledge for ensuring crop yield and food quality as well as implementing soil phytoremediation.

## 1 Introduction

The simultaneous presence of polycyclic aromatic hydrocarbons (PAHs) and heavy metals in soil and aquatic habitats has become more prevalent, as both pollutants originate from analogous anthropogenic activities, such as waste incineration, vehicle emissions, and power generation (Deng et al., 2018; Zwolak et al., 2019; Hu et al., 2020; Zhang et al., 2020). Among the 16 PAHs pollutants of priority concern, pyrene has gained attention due to its high phytotoxicity, mutability, and widespread presence in environments (Gabriele et al., 2021). Besides, the multiple sources, high persistence, and non-degrading nature of heavy metals, including cadmium (Cd), exert an accumulative effect on plant growth from seed germination to fruition (Zhou et al., 2021; Wu et al., 2022). Despite the fact that copper (Cu) is an essential micronutrient to bionts, both plants and humans are increasingly at risk of excessive intake due to heightened Cu contamination in soil and water, which subsequently enters the food chain (Fsanz, 2003; Fao/Who, 2011; Kumar et al., 2021), causing a harmful effect on the ecosystem and posing a risk of carcinogenic, teratogenic, and mutagenic disorders to human health (Moscoso et al., 2012; Deng et al., 2018).

While the potential for pollutants extraction by plants provides the possibility for soil phytoremediation, significant reduction of vegetation biomass and quality due to contamination causes worldwide concerns (Gao and Zhu, 2004; Peng et al., 2017; Deng et al., 2018; Sufian et al., 2022). Maize (*Zea mays* L.) is widely cultivated cereal crop, possessing comparatively high biomass yields, fast growth and tolerance to abiotic stress. These traits make it a valuable model for assessing the impact of various contaminants on plant growth and pollutant accumulation (Li et al., 2017; Abdelgawad et al., 2020). Both PAHs and heavy metals have been found to produce growth inhibition and pollutant accumulation in maize, especially with the increase in contaminated levels (Lin et al., 2008; Houshani et al., 2019). In addition, the interaction between PAHs and heavy metals under joint stress could generate more complicated effects on plant growth and physiology (Lu et al., 2014). The occurrence of organic pollutants could reduce the phytotoxicity of metals via chelation but increase their bioavailability and accumulation in plants (Ma et al., 2016; Ren et al., 2019). Conversely, the presence of heavy metals disturbs the root physiology and alters the rhizosphere bacterial community (Wang et al., 2021), which could change the biodegradation of PAHs and their subsequent impacts on plant growth. These interactions exert either synergistic (Deng et al., 2018) or antagonistic (Lin et al., 2008; Lu et al., 2014) effects on plant growth.

To overcome the abiotic stress, plants adopt protective strategies such as antioxidant enzyme production, pollutant chelation or sequestration, and regulation of biomass allocation (Yergeau et al., 2018; Thakur et al., 2020). Deng et al. (2018) found that while the presence of Cu/Pb/Cr strengthened the root and shoot biomass of pak choi (*Brassica chinensis L.*) compared to single PAHs stress, co-existence of PAHs and Cd/Zn significantly reduced the gross biomass. Lin et al. (2008) demonstrated that the root growth of maize was reduced under pyrene-Cu co-contamination, but the shoot growth was improved except for a high concentration of Cu (400 mg/kg) and pyrene (500 mg/kg). In contrast, the growth inhibitory effect was more significant in the aboveground part than the root part when Fire Phoenix (a mixture of *Festuca L.*) was planted in pyrene-Cd contaminated soil (Dai et al., 2020), as the presence of metals seriously damaged the ultrastructure in leaves and thus declined the photosynthetic pigment levels (Sorrentino et al., 2018). Li et al. (2021) also determined the increased lipid peroxidation in willow (*Salix* sp.) leaves under pyrene-Cd joint stress. A study by Song et al. (2022) suggested the synergetic toxicity of PAHs-Cd on the growth and physiology of Bermuda grass (*Cynodon dactylon (L.) pers.*) as the antioxidant enzyme activity and lipid peroxidation were significantly increased compared to single PAHs or Cd stress. Accordingly, plant physiological and growth inhibition and their self-defense mechanism vary with plant species and their cultivars, as well as the concentration and combination of contaminants. Considering the importance of maize crop, it is necessary to thoroughly investigate the growth and physiological response in the light of photosynthesis, lipid peroxidation, and enzyme activities under interactive effects of pyrene, Cu, and Cd. Also, there is a need to explore the capacity of plant defense systems by categorizing the growth inhibitory effects.

The current study aimed to investigate the maize physiological and growth response under the different concentrations and combinations of pyrene, Cu, and Cd, with an indicator adopted and further developed to identify the joint stress on growth inhibition. The findings provide valuable knowledge for the favorable production of maize crop (both in yield and quality) and for the implementation of phytoremediation technologies in PAHs-heavy metals co-contaminated soils.

## 2 Materials and methods

### 2.1 Maize culture

Maize seeds were germinated for one week, and the seedlings with a 12-14 cm height were selected for pot-culture. Agricultural soil was used for experiments, with a pH of 8.0 and an organic matter content of 17.6 g/kg. Subsequent to the elimination of stone and plant debris, the soil sample was air-dried and subjected to a 2-mm screen prior to utilize. Each 1 kg of soil was moved into a plastic pot (16cm diameter × 14cm height). The tested contamination groups and detailed soil treatment have been described in Wang et al. (2021). The evaluated levels of pyrene, Cu and Cd contamination were 5, 50, 400, and 750 mg/kg soil; 100, 500, 800, and 1000 mg/kg soil, and 1, 20, 50 and 100 mg/kg soil, respectively. Pyrene of analytical reagent (AR) grade (≥ 98.0%; Shanghai Macklin Biochemical Co., Ltd.) was solubilized in 100 mL of acetone (≥ 99.5%, AR) and then applied to the specified soil samples by spraying. Similarly, solutions with specific Cu/Cd concentrations were added to the soils. In addition, four groups of joint contamination were examined (**Table 1**). The unspiked soil was served as a control (CK). Fertilizers containing 95 mg/kg of N from CO(NH_2_)_2_, 480 mg/kg of P_2_O_5_, and 318 mg/kg of K_2_O were incorporated into the soil for maize cultivation. After soil incubation for 20 days, the pre-germinated maize seedlings were planted in the pots. Plant cultures were piloted in triplicate for each treatment within three 1000-L phytotrons (1.25 m × 0.65 m × 1.90 m, SGZ-1000A, Hangzhou Shuolian Instrument Co., Ltd) for a duration of 57 days. The soil moisture was kept at 70% of field capacity by making adjustments to the water levels every 24 hours. The temperature reached 30°C during daytime (16 h) and 25°C at night (8 h), with 6 h of light daily and a relative humidity of 80%.

**Table 1:** Tested groups and contaminated levels in soils.

Tested groups		Contaminated levels			
		I	II	III	IV
**Control**	–	CK			
**Single contamination**	Pyrene	P5**^i^**	P50	P400	P750
	Cu	Cu100	Cu500	Cu800	Cu1000
	Cd	Cd1	Cd20	Cd50	Cd100
**Joint contamination**	Pyrene + Cu	P5Cu100	P50Cu500	P400Cu800	P750Cu1000
	Pyrene + Cd	P5Cd1	P50Cd20	P400Cd50	P750Cd100
	Cu + Cd	Cu100Cd1	Cu500Cd20	Cu800Cd50	Cu1000Cd100
	Pyrene + Cu + Cd	P5Cu100Cd1	P50Cu500Cd20	P400Cu800Cd50	P750Cu1000Cd100

^i^ P represents pyrene, and the following number means the contaminated concentration at mg/kg soil.

### 2.2 Determination of plant physiology

#### 2.2.1 Biomass and cell viability

After 57-day culture, plant height (for the aboveground part) was measured. Then the plants were extracted from the pots, rinsed with deionized (DI) water to eliminate soil particles, and subjected to air-dry. Shoots and roots were segregated, and their fresh weights were quantified. Root section samples were dyed with 0.5% Evans blue solution for 1 min, rinsed with DI water and photographed using a microscope (XSP-35TV, ×1600, Phenix Optics Co., Ltd., China) to determine the cell viability.

#### 2.2.2 Chlorophyll-a

Chlorophyll-a (Chl-a) was extracted by acetone (Zhang et al., 2009). Fresh leaves were cut into pieces, and 0.3 g was moved into a 10-mL centrifuge tube containing 3 mL acetone. The mixtures were ground by a homogenizer in an ice-bath, added with 5 mL acetone solution (80%), and centrifuged at 4000 rpm for 10 min. Then the supernatant liquor was diluted with 80% acetone solution and determined using a UV-spectrophotometry (EU-2600R, Shanghai Onlab Instrument Co., Ltd, China) at 645 nm and 663 nm, with comparison to 80% acetone blank.

#### 2.2.3 Lipid peroxidation

Malondialdehyde (MDA) was determined with thiobarbituric acid (TBA) to analyze the lipid peroxidation (Zhang et al., 2009). The enzyme was extracted from 0.5 g leaf sample by grinding with 5 mL 10% trichloroacetic acid (TCA). After 10-min centrifugation at 4000 rpm, 2 mL supernatant liquor was mixed with 2 mL 0.5% TBA, bathed in boiling water for 20 min, cooled down rapidly, and centrifuged at 3000 rpm for 10 min. Spectrophotometry was performed to determine MDA at 450 nm, 532 nm, and 600 nm.

#### 2.2.4 Antioxidant enzymes

The antioxidant enzymes including peroxidase (POD) and superoxide dismutase (SOD) were determined using the enzyme-linked immunosorbent assay kits (Shanghai Meilian Biotechnology Co., Ltd., China). Fresh leaves were cut into pieces, ground with liquid nitrogen, and centrifuged at 6000 rpm for 15 min with 5 mL phosphate buffer. The supernatant liquor was sampled and determined using a microplate spectrophotometer (BioTek Instruments, Inc., USA).

### 2.3 Determination of pollutant accumulation

The accumulation of pyrene, Cu, and Cd in maize plants after 57-day exposure was determined according to Wang et al. (2021). Briefly, the lyophilized powder samples (0.2 g each from root, stem, and leaf) were soaked in 1:1 hexane-acetone solution (20 mL) and ultra-sonicated. The extract was then centrifuged and purified, and the pyrene concentration was determined by high-performance liquid chromatography (HPLC). For Cu and Cd accumulation, the powder sample (1 g) was soaked in 1:2 HClO_4_-HNO_3_ (15 mL) for 8 h, thereafter heated on a hot plate until the cessation of brown vapors, and then re-heated following the addition of 5 mL HNO_3_. The resultant solution was subsequently filtered and studied with a spectrophotometer for atomic absorption (TAS-986, Thermo Fisher Scientific, USA). The determination precision was validated by comparing with the authorized standard material, shrub leaves and branches GBW07603 (National Standard Substances Center of China). The result of pyrene, Cu, and Cd accumulation has been analyzed in detail in Wang et al. (2021), thus it is described with the discussion of maize growth and physiology in this article.

### 2.4 Data processing

The joint toxicity of pyrene and heavy metals on maize growth was examined according to the Abbott’s formula (Gisi, 1996; Teisseire et al., 1999; Chesworth et al., 2004; Gatidou et al., 2007). The expected combined inhibition $C_{exp}$ is usually calculated using Eq. (1) when exposed to joint stress. Considering three contaminants tested in this study, Eq. (2) is developed for the expected inhibition.


(1) $$C_{exp}=I_{1}+I_{2}-I_{1}I_{2}$$

(2) $$C_{exp}=I_{1}+I_{2}+I_{3}-I_{1}I_{2}-I_{1}I_{3}-I_{2}I_{3}+I_{1}I_{2}I_{3}$$

where $I_{1}$ , $I_{2}$ and $I_{3}$ were the inhibitions caused by relevant single contaminations.

The indicator of combined contamination, ratio of inhibition *RI*, which compares the observed inhibition *OI* and the expectation $C_{exp}$ , was assessed using Eq. (3). *RI* > 1 indicates synergism, *RI* = 1 means simple additivity, and *RI* < 1 represents antagonism (Gisi, 1996; Teisseire et al., 1999; Chesworth et al., 2004; Gatidou et al., 2007).


(3) $$RI=OI/C_{exp}$$

The experimental results are presented as the mean ± standard deviation (SD) of triplicates. The analysis of variance (ANOVA) was performed with SPSS 22.0 to evaluate the significance of difference. In addition, multivariate statistical techniques, including the Pearson’s correlation analysis and redundancy analysis (RDA), were adopted to further explore the effects of contaminant accumulation on plant growth and physiological response.

## 3 Results

### 3.1 Cell viability

**Figure 1:**
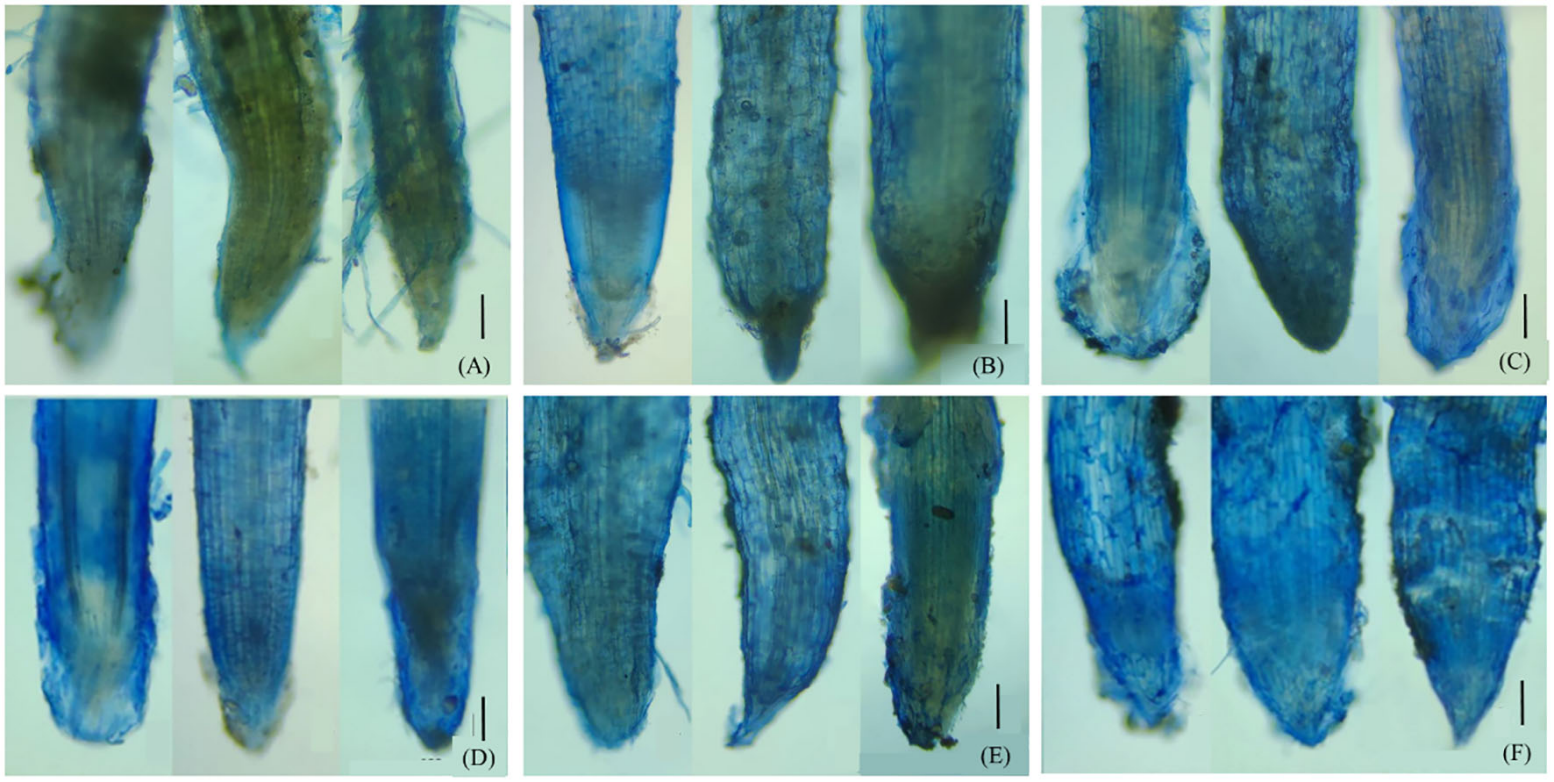
Dyeing of the maize roots by Evans blue: **(A)** CK, **(B)** P750, **(C)** Cu1000, **(D)** Cd100, **(E)** P750Cu1000, and **(F)** P750Cd100. The solid black lines represent a reference to 0.5 mm scale.

Evans blue, a kind of azo dye, can distinguish the dead cells from the live ones. The surface layer of maize roots grown in unspiked soil appeared light blue (**Figure 1A**), indicating the presence of necrotic cells. The dyed blue was much more pronounced in P750 (**Figure 1B**) and Cu1000 (**Figure 1C**) compared to CK. By contrast, joint contamination of pyrene and Cu (**Figure 1E**) did not produce a noticeable difference compared to the single treatments. Physio-toxicity caused by Cd and PCd contaminations at level IV appeared to be more significant, given the deeper blue of root cells (**Figures 1D, F**).

### 3.2 Plant biomass

#### 3.2.1 Single contamination

**Figure 2:**
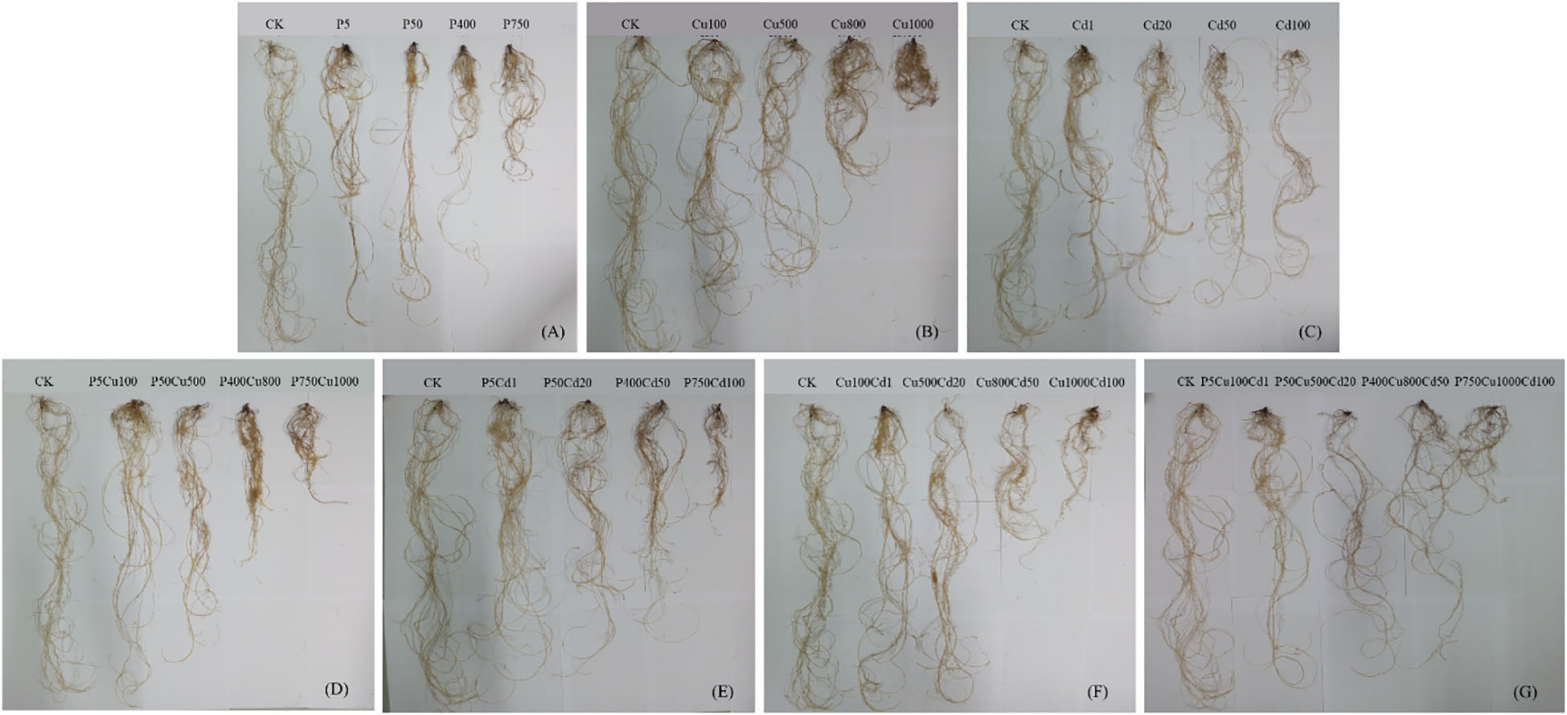
Maize roots after 57-day culture under single contaminations of **(A)** pyrene, **(B)** Cu, and **(C)** Cd and joint contaminations of **(D)** pyrene-Cu, **(E)** pyrene-Cd, **(F)** Cu-Cd, and **(G)** pyrene-Cu-Cd.

**Figure 3:**
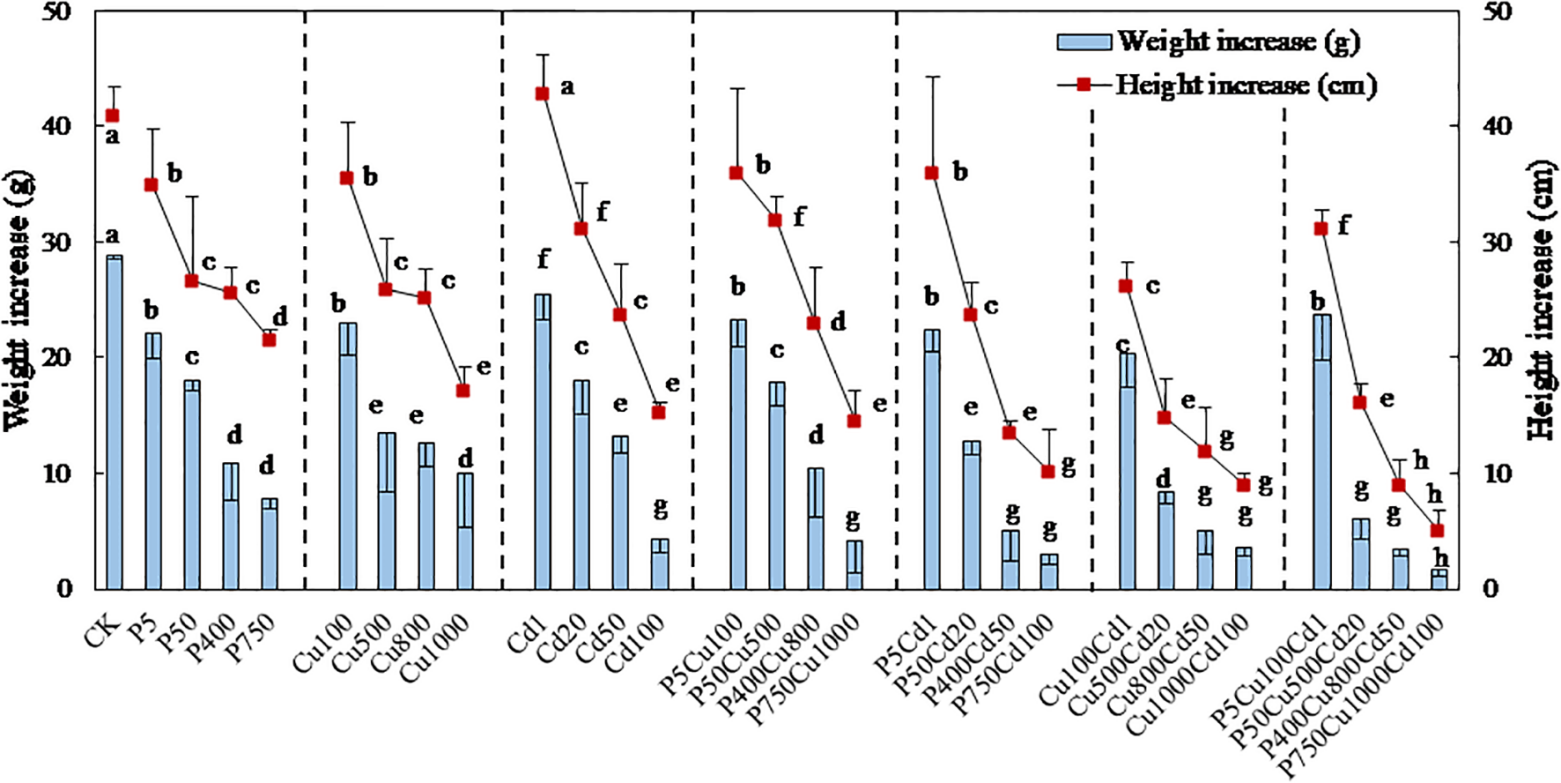
Increases in height and fresh weight of maize plants exposed to varying levels of pyrene, Cu, Cd, and the associated combinations for 57-day culture. The results are expressed as the mean ± SD of triplicates, with distinct letters denoting significant difference at *p* < 0.05 level.

For 57 days of culture, the maize biomass was significantly reduced under single and joint treatments with pyrene, Cu, and Cd (**Figures 2**, **3**). Increased contaminant concentrations (levels I-IV) decreased plant height and fresh weight. **Supplementary Figure S1** and **Figure 2** show the maize plants and roots after 57-day culture, respectively, while **Figure 3** presents the increase in plant height and fresh weight. Exposed to individual pyrene, Cu, and Cd contaminated soil, the increase in fresh weight was reduced by 23-73%, 20-65%, and 12-85%, respectively, compared to CK (**Figure 3**). **Supplementary Figure S2** further shows the fresh weight of maize roots, stems, and leaves separately. It appeared that at contaminated levels I-II, pyrene, Cu, and Cd contaminations produced a more substantial impact on leaves, stems, and roots of maize biomass, respectively, whereas this oriented inhibition disappeared as the contaminations increased to levels III-IV. Comparably, the increase in plant height was reduced by 14-47% (pyrene) and 13-58% (Cu), which was almost similar for levels II and III. For Cd spiked soil, plant height was slightly improved at Cd1 condition compared to CK, while continually decreased up to 63% with increasing contaminated level.

#### 3.2.2 Joint contamination

For the joint contaminations, the fresh weight increase of plants was reduced by 19-86% (PCu), 22-90% (PCd), 30-88% (CuCd), and 18-94% (PCuCd) compared to CK at contaminated levels I-IV. Meanwhile, the reduction in height increase ranged from 12-65%, 12%-75%, 36-78% and 24-88% for PCu, PCd, CuCd and PCuCd, respectively. However, the growth effect under the joint stress of contaminants was complex and related to the combination of contaminants and their concentration levels. The PCu co-contamination showed no obvious influence on the maize growth for level I compared to individual pyrene or Cu treatment (**Figure 3**). By contrast, the increase in plant height at level II was elevated by 19% and 23% compared to P50 and Cu500, respectively. Meanwhile, the fresh weight of maize shoots was significantly increased by joint contamination (**Supplementary Figure S2**), leading to a 39% increase in total weight gain in P50Cu500 compared to Cu500 (**Figure 3**). Nevertheless, the growth inhibition was gradually enhanced, with the *RI* increased from <0.5 at level I to ~0.9 at level IV (**Table 2**). In other words, the antagonistic effect of PCu co-contamination on maize biomass at low levels was escalated to additive inhibition (RI ≈ 1) as pollutant concentrations increased.

**Table 2:** The ratio of inhibition of joint contamination on maize growth.

	Contaminations				
	Levels	PCu	PCd	CuCd	PCuCd
**Weight increase**	I	0.49	0.70	1.00	0.39
	II	0.54	0.91	1.01	0.97
	III	0.76	1.00	1.03	0.95
	IV	0.94	0.93	0.92	0.96
**Height increase**	I	0.47	-**^i^**	–	–
	II	0.38	0.84	1.23	0.88
	III	0.71	1.05	1.10	1.00
	IV	0.83	0.94	0.93	0.96
**Chl-a**	I	–	–	0.79	–
	II	0.18	1.49	0.88	0.92
	III	0.45	0.61	0.84	0.76
	IV	0.53	0.55	0.96	0.96

^i^Height increase and Chl-a under contaminated level I were not significantly different from the controls and, therefore, some inhibitions (*I*_1_, *I*_2_, *I*_3_ or *OI*) appeared to be negative, making Eq. (1) and Eq. (2) not applicable.

For PCd co-contamination at level I, the plant growth resembled that seen to individual pyrene treatment (**Figure 3**), indicating that pyrene co-presence prevails normal growth in this low-contamination scenario, potentially mitigating the irregularities induced by Cd contamination. However, maize biomass rapidly declined with increasing contaminated levels, and the *RI* values were close to 1.0 at levels III and IV. The combined effect of PCd co-contamination appeared to be additive at high contaminated levels, implying that the inhibition of maize growth was aggravated.

Unlike the above, when exposed to the combined stress of heavy metals Cu and Cd, maize growth was significantly reduced at any contaminated level (**Figure 3**). The *RI* values (**Table 2**) show that the joint stress of Cd and Cu inhibited the maize growth with an additive or even synergetic effect in many cases. Exposed to PCuCd co-contamination at level I, plant growth was improved in terms of fresh weight (17%) and height (19%) increase compared with CuCd co-treatment, resulting in an antagonistic effect with *RI* = 0.39. It implies that pyrene could somewhat diminish the stress of heavy metals at low concentrations. In contrast, increasing contaminations significantly suppressed maize growth, and the inhibition appeared purely additive for levels II-IV. **Supplementary Figure S2** shows that CuCd and PCuCd co-contaminations produced a more significant impact on maize shoots rather than roots.

### 3.3 Chl-a

**Figure 4:**
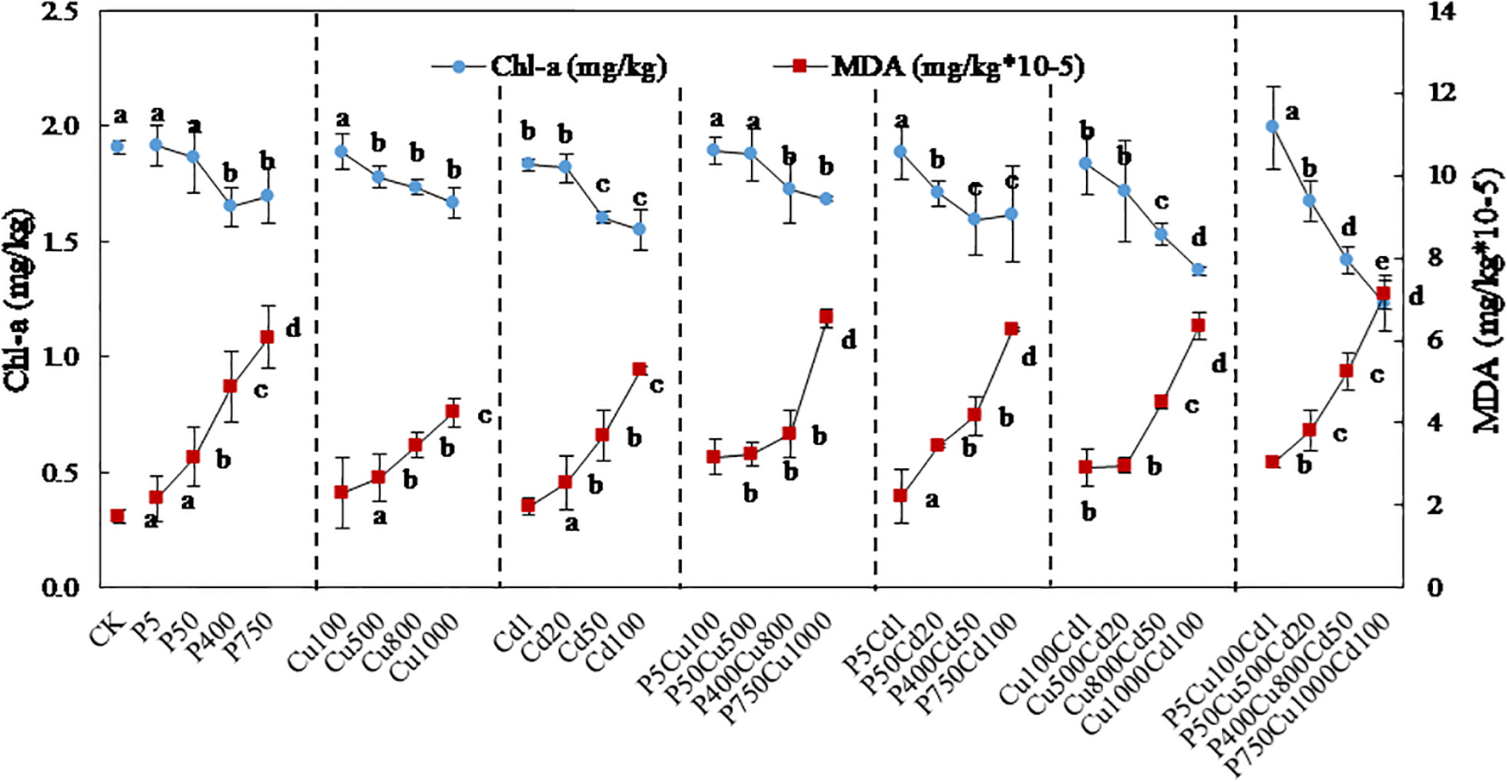
Profiles of Chl-a content and MDA formation in maize leaves exposed to varying levels of pyrene, Cu, Cd, and the associated combinations for 57-day culture. The outcomes are expressed as the mean ± SD of triplicates, with distinct letters denoting significant difference at *p* < 0.05 level.

The Chl-a content in plant leaves decreased with the increasing contaminations under single and joint stress of pyrene, Cu, and Cd (**Figure 4**). Compared with CK, single pyrene or Cu treatment at level I did not significantly influence the Chl-a content. As the contaminant concentrations increased, the Chl-a content gradually reduced, and the inhibition reached 14% for P400 and 13% for Cu1000. Likewise, the Chl-a content was reduced by 4-19% when exposed to single Cd stress. Recognizable chlorosis can be observed for Cu and Cd treatments at level IV (**Supplementary Figures S1B, C**).

For the joint contaminations, PCu produced a similar effect on Chl-a compared to single pyrene or Cu treatment. Generally, PCu and PCd co-contaminations produced an antagonistic effect on Chl-a, with the *RI* ≤ 0.6 (**Table 2**). An *RI* = 1.5 was obtained at P50Cd20 because the inhibitions caused by P50 and Cd20 were slight, resulting in a small $C_{exp}$ in Eq. (3). When exposed to low contamination (level I), the Chl-a content was increased by 3% (PCd) and 9% (PCuCd) compared to Cd and CuCd, respectively. It indicates that the photosynthetic activity was amended in the presence of pyrene at a low level. In contrast, the inhibition of Chl-a reached up to 28% (CuCd) and 35% (PCuCd) at level IV compared to CK, with an additive effect on photosynthetic pigmentation. Plants exposed to this stress present severe chlorosis in the leaves (**Supplementary Figures S1F, G**).

### 3.4 MDA

The MDA content, an indicator of lipid peroxidation, greatly increased with increasing concentrations of pyrene, Cu, and Cd (**Figure 4**). Single contaminant stress increased the MDA content in maize leaves from 1.7×10^-5^ mg/kg (CK) to 6.1×10^-5^ mg/kg (P750), 4.2×10^-5^ mg/kg (Cu1000), and 5.3×10^-5^ mg/kg (Cd100), respectively. At contaminated level I, there was no obvious change with single contaminations compared to CK, but a noticeable increment can be determined under co-contamination treatments. MDA concentrations were increased up to 3.6-4.1 times under joint contaminations at level IV, with the maximum impact determined at P750Cu1000Cd100.

### 3.5 Antioxidant enzymes

**Figure 5:**
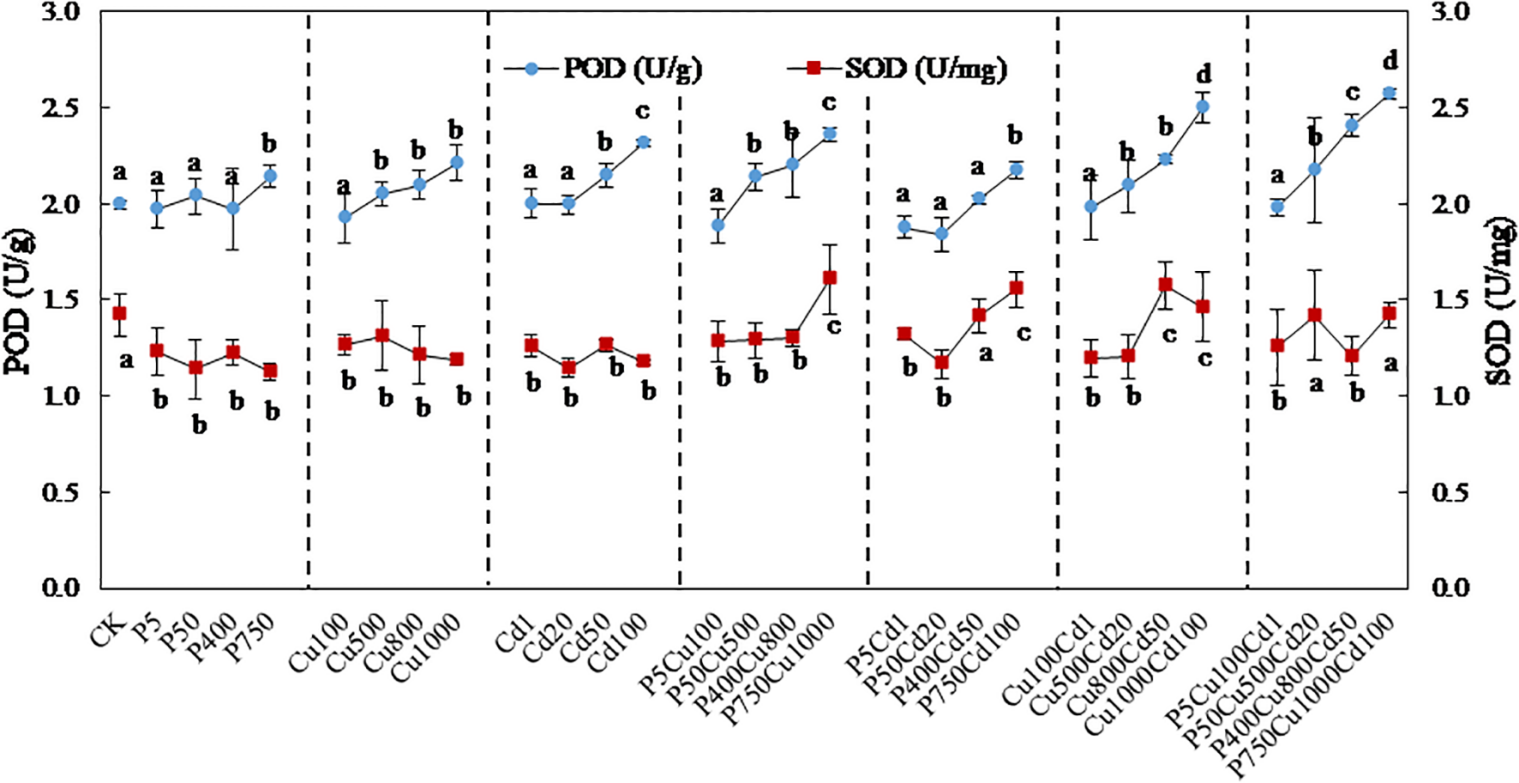
The variation in POD and SOD activity in maize plants exposed to varying levels of pyrene, Cu, Cd, and their combinations for 57-day culture. Data are presented as the mean ± SD of triplicates, with different letters denoting significant difference at *p* < 0.05 level.

The POD activity showed no significant variation at the low contamination level of all treatments (**Figure 5**). With the increasing contaminated levels, the POD activity was gradually elevated. The effect of joint contamination was more significant compared to single stress, with the maximum (1.3 times of CK) determined at Cu1000Cd100 (level IV) and P750Cu1000Cd100 (level IV).

By contrast, the SOD activity was reduced from 1.4 U/mg to 1.1-1.3 U/mg under single contaminations, which showed no significant variation associated with contaminant concentrations. Joint contaminations at low/medium levels produced a similar effect to the single treatments, whereas a high co-contaminated level increased the SOD activity to 1.5-1.6 U/mg.

### 3.6 Effects of pyrene, Cu, and Cd accumulation in maize

Along with the exposure, pyrene, Cu, and Cd accumulated in varying degrees in maize plants (**Supplementary Figure S3**). Pyrene concentration in maize plants was negligible (< 10 mg/kg) in most cases. However, co-presence with Cu at III-IV levels dramatically increased the pyrene accumulation (30-100 mg/kg) in maize roots. As for the metals, Cu and Cd contents in plants consistently rose with increasing degrees of contamination, which were mainly trapped in maize roots. In addition, the joint contamination tended to increase the Cu accumulation but decrease Cd in maize shoots.

**Figure 6:**
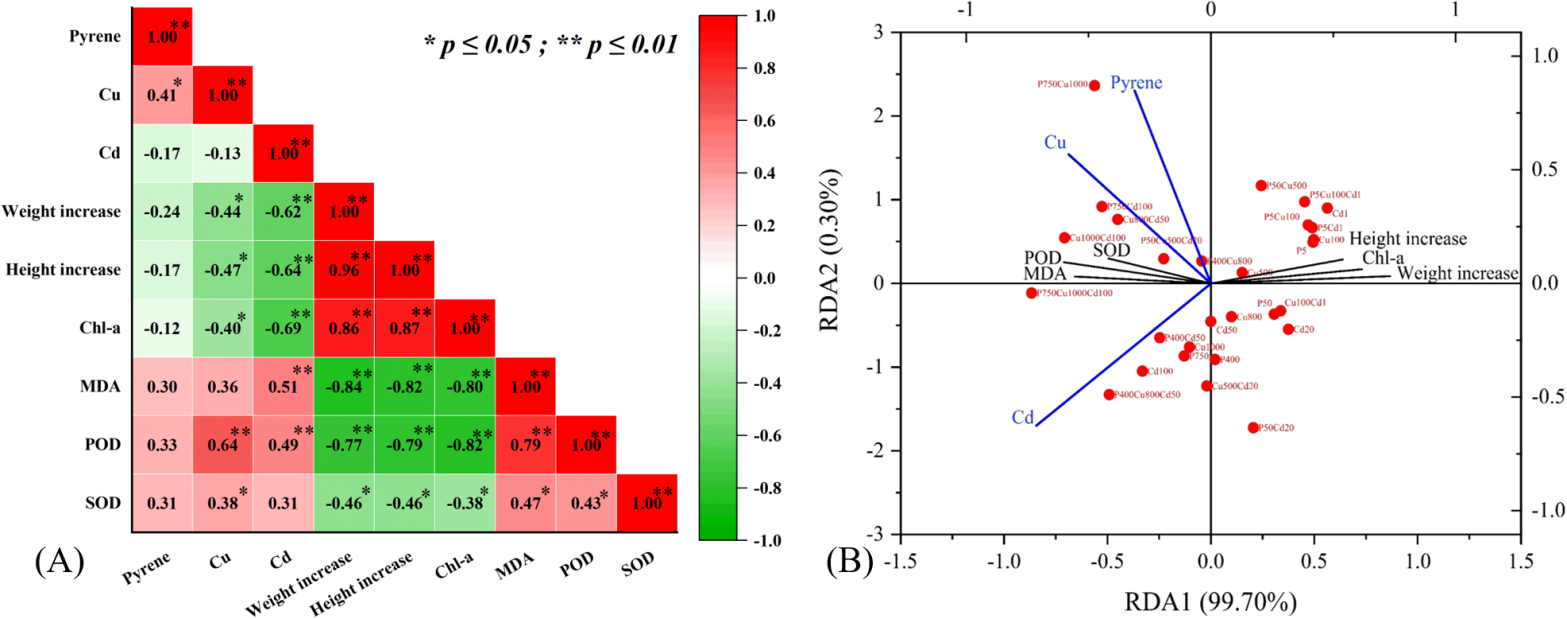
The effects of pyrene, Cu, and Cd stress on maize plant growth: **(A)** correlation matrix with significant correlations values denoted s * P ≤ 0.05, ** P ≤ 0.001 and **(B)** RDA of physiological characteristics with contaminant accumulation.

The contaminant accumulation produced a negative effect on plant biomass increase and photosynthesis (Chl-a) and a positive influence on lipid peroxidation (MDA) and antioxidant enzymes (POD and SOD) (**Figure 6A**). Generally, the significance of effects appeared Cd (*p < 0.01*) > Cu (*p < 0.05*) > pyrene (*p > 0.05*), except for the antioxidant enzymes which were more significantly stimulated by Cu. The RDA biplot further illustrated the correlation between stress and plant physiological response (**Figure 6B**). For physiological parameters, biomass and Chl-a positively associated with x-axis while MDA, POD, and SOD negatively correlated, representing a differentiation of samples with varying contaminated levels. For contaminant indicators, pyrene, Cu and Cd were projected on the opposite with y-axis, differentiating the tested treatments. In other words, maize growth and physiological characteristics were impacted both by the contaminant types and stress levels.

## 4 Discussion

### 4.1 Maize growth response to pyrene stress

Maize growth showed a certain tolerance and adaptive capacity to low-level (< 50 mg/kg) stress induced by pyrene, reflected by the plant appearance and relative stability of leaf physiology. Similar findings have also been reported for the willow that addition of 15.72 mg/kg pyrene slightly decreased the biomass with negligible change in other physiological parameters (Li et al., 2022). This is partially because that the majority of pyrene in soil can be dissipated via volatilization, photodegradation, or rhizosphere biodegradation during plant culture, with only a small part accumulated in maize plant (Wang et al., 2021). Even low-level organic contaminants could promote the growth of some specific plants. For example, Yang et al. (2022) and Sun et al. (2011) have observed a stimulating effect on plant growth (*Brassica campestris* L. and *Tagetes patula*) induced by <50 mg/kg pyrene and benzo[a]pyrene, respectively.

However, maize growth and the physiological regulations were significantly impacted with an elevated pyrene level. Exposed to 750 mg/kg pyrene treatment, maize roots showed a significant deleterious impression (**Figures 1B**, **2A**), as the rhizosphere-promoted degradation was inhibited under high-level pyrene stress (Wang et al., 2021). Although most of the pyrene was vanished eventually, the stress elevated the level of reactive oxidative species, which can reduce photosynthesis by deteriorating protein complex embedded in chloroplast membrane (Liu et al., 2009; Zhang et al., 2013). Consequently, the potential in water and nutrients uptake from soils by root system was shrunk (Lu et al., 2014), leading to a significant decrease in plant biomass.

### 4.2 Phytotoxicity of heavy metals to maize

Plant cells adopt homeostasis to regulate the concentrations of metal ions and maintain a stable biochemical environment, especially at level I with single metal stress (**Figures 3**, **4**). However, this homeostatic mechanism becomes supersaturated under excessive metal stress in terms of elevated concentrations and metals co-existence, and failed to ensuing the toxicological processes (Colin et al., 2012). Thus, the exposure of Cu (redox-active) and Cd (redox-inactive) transition metals catalyzes the formation of reactive oxygen species such as free radicals (**Figure 5**) (Rahman and Singh, 2019; Abdelgawad et al., 2020). Lipid peroxidation induced by these free radicals accelerates MDA production, which exhibits cytotoxicity to damage proteins, pigments, and nucleic acids (Tanyolac et al., 2007; Ekmekçi et al., 2008).

Accordingly, the overexpression of antioxidant enzymes activity in plants has been reported to overcome the Cu or Cd toxic effect (Liu et al., 2017). For example (Anwar et al., 2019; Franco et al., 2022), and (Anwar et al., 2019; Franco et al., 2022) observed an increase in POD and SOD activity in maize when exposed to 0-30 mg/kg Cd or 0.05-100 μM Cu stress. However, the opposite pattern was also determined, as Zhang et al. (2019) reported that the POD and SOD activity in two maize cultivars decreased at 50 mg/kg Cd stress compared to the uncontaminated case. Tanyolac et al. (2007) found that exposure to 0.5 mM Cu produced no significant change in POD activity but slightly increased SOD activity in maize leaves, while increasing Cu concentration to 1.5 mM significantly increased the activity of both. Actually, the response of antioxidant enzymes activity depends on maize cultivars (Ekmekçi et al., 2008), growth status and contaminated levels (Yang et al., 2021). According to the results, maize cultivar used in this study exhibited a comparatively satisfactory tolerance against Cu or Cd stress with the relative stability of MDA content at low Cu (< 500 mg/kg) and Cd (< 20 mg/kg) concentrations. Nevertheless, increasing contamination levels significantly affect many metabolism pathways (e.g., carbohydrates and energy) and cellular processes (e.g., transport and catabolism) (Wang et al., 2021), which obstructed the transportation of Fe and the synthesis of photosynthetic pigments (Sorrentino et al., 2018), resulting in a reduction of plant biomass and the chlorosis.

Worse still, the contaminated soil in practice usually contains multiple metals, which exert complicated effects on the cultivated plants. The co-occurrence of Cu and Cd produced more severe growth inhibition than its single stress across all contaminated levels in this study, with the combined inhibition appearing as a pure additive or even synergetic. While the presence of Cd reduced Cu levels in maize roots, it enhanced Cu transfer from roots to shoots, here promoting Cu accumulation in the aerial portions (Wang et al., 2021), thus increasing the toxicological effect on the plants. Meanwhile, the co-stress of Cu and Cd further disturbed the physiological functions (including biosynthetic and metabolic reactions) in maize roots and rhizosphere microbial communities (Wang et al., 2021), impeding the cellular tolerance to metal stress. Qian et al. (2009) also reported that *Chlorella vulgaris* growth was synergistically inhibited due to decreased cell growth and Chl-a level, blocked electron transport and CO_2_ assimilation, and increased reactive oxygen species under the joint stress of Cu and Cd. In addition, cytological analysis of cardoon cultivars grown in Cd-Pb polluted soil has demonstrated the destruction of chloroplasts under joint stress (Sorrentino et al., 2018). These ultrastructural damages directly impeded photosynthesis and nutrient absorption in association with stomatal conductance, consequently inhibiting plant growth. Premature senescence (e.g., chlorosis and necrosis) of leaves can be the typical symptom of these effects (**Supplementary Figure S1**).

### 4.3 Joint effects of pyrene and heavy metals on maize

The interaction of pyrene and heavy metals in soil seemed to be associated with their contaminated levels, which caused complex effects on maize growth. Generally, the co-occurrence of pyrene and Cu/Cd at levels I-II produced no obvious difference in maize growth compared to its respective single contaminations, with exposure to P50Cu500 even slightly increased the plant biomass compared to P50 and Cu500. The presence of some specific metals is beneficial to the rhizosphere biodegradation of organic pollutants as they decrease the abundance of metal-sensitive bacteria and mitigate nutrient competition (Xu et al., 2018; Wang et al., 2021). Conversely, a low-level pyrene addition can reduce the metal phytotoxicity to a certain extent via the formation of metal-organic chelates (Ma et al., 2016; Ren et al., 2019), which exerts gentler stress on root physiology. The presence of 5mg/kg pyrene alleviated the negative effect on Chl-a formation induced by Cd1 or Cu100Cd1. Similar pattern has also been observed in willow and the increased photosynthetic activity was ascribed to the promoted nutrient uptake (Li et al., 2022). In addition, moderate pyrene concentration could be a potential carbon source for the prosperity of biodegrading microbes in soil and stimulate the secretion of phytohormones to overcome metal stress (Weyens et al., 2009). Consequently, the joint stress of pyrene with metals appeared antagonistic under these circumstances. Likewise, it has been demonstrated that the co-presence of pyrene-Cu in maize (Lin et al., 2008) and pyrene-Cu/Cd in tall fescue (Lu et al., 2014) showed improvement in biomass along with the increased accumulation of metals in plant organs. This implies that specific organic agents can be adopted to aid in the phytoremediation of metal-contaminated soil without reducing the biomass of hyperaccumulators, on condition that the contaminant properties, agent dosage, and plant species are comprehensively considered.

Nevertheless, the severity of antagonism on maize growth intensified as the co-contaminated level increased, with the negative effects on biomass and leaf physiology aggravated (**Table 2**). The intensive metal stress induced oxidative damage in plant cells, disturbed the metabolic process, and inhibited the biodegradation of pyrene (Kang et al., 2010; Toyama et al., 2011; Mcmanus et al., 2018; Sorrentino et al., 2018). Besides, the biodegradation of organic contaminants was hindered due to changes in root exudates and heavy metal contamination, which affected rhizosphere microbial populations and activity, thereby hindering the biodegradation of organic contaminants (Chigbo et al., 2013; Lu et al., 2014; Liu et al., 2017; Jiang et al., 2019; Gabriele et al., 2021). It elucidates the elevated pyrene accumulation in maize roots under pyrene and Cu co-contamination (**Supplementary Figure S3**). Sun et al. (2011) observed that while Cu at a low level (100 mg/kg) did not affect plant development compared to single benzo[a]pyrene stress, an increased Cu level to 500 mg/kg significantly lowered uptake of benzo[a]pyrene and targeted the biomass of *Tagetes patula*. On the other hand, the co-presence of excessive organic pollutants disrupted the metabolic processes, molecular-binding functions, and catalytic activities of enzymes in plant roots (Zhang et al., 2013; Wang et al., 2021), thus reducing the cell tolerance to metal stress. Lin et al. (2006) found that with the initial pentachlorophenol (PCP) concentration of 50 mg/kg, the growth of *Lolium perenne* L. and *Raphanus sativus* was enhanced with increasing Cu level (0-300 mg/kg), whereas an adverse effect was observed with initial PCP of 100 mg/kg. This suggests that the plants reach their detoxification capacity and degradation limits under intense joint stress, which provides important information for protecting food crop safety and formulating phytoremediation approaches using suitable hyperaccumulators. To acquire a better understanding of how maize reacts to co-contaminants, future research should look at the molecular and biochemical mechanisms involved, with an emphasis on antioxidant enzyme control and chlorophyll stability.

## 5 Conclusions

Pot tests were conducted to explore the maize growth and physiological response to single and joint stress of pyrene, Cu, and Cd. The application and development of growth inhibition indicator further contribute to the assessment of combined contamination. Generally, the growth inhibition produced by Cd (50-100 mg/kg) was stronger compared to Cu (800-1000 mg/kg), associated with the lethal effect of Cd on root cells. Joint stress of Cu and Cd further aggravated the adverse effects and generated synergetic inhibition, and the presence of pyrene (400-750 mg/mg) with such metal stress levels produced additive inhibition on maize growth. The maize cultivar used in the current study showed a satisfactory tolerance of a low-level pyrene and/or metal stress, with the relatively stable Chl-a content and antioxidant enzymes (POD and SOD) activity. Nevertheless, the photosynthesis and antioxidant system were significantly damaged with increasing contaminant concentrations, resulting in chlorosis and biomass reduction. These findings assist in protecting crop yield and food quality supporting soil phytoremediation, and underscoring the need for further research on metabolic pathways involved in maize response to co-contaminants.

## Data Availability

The original contributions presented in the study are included in the article/**Supplementary Material**. Further inquiries can be directed to the corresponding author.
