# Supplementary material for: Single/joint effects of pyrene and heavy metals in contaminated soils on the growth and physiological response of maize (Zea mays L.)
Source: Front Plant Sci. 2024 Dec 2;15:1505670. doi: 10.3389/fpls.2024.1505670 (PMC11648570; doi:10.3389/fpls.2024.1505670)
Supplement: Supplementary file 1 [file DataSheet1.pdf]

Supplementary Material

1 Supplementary Figures

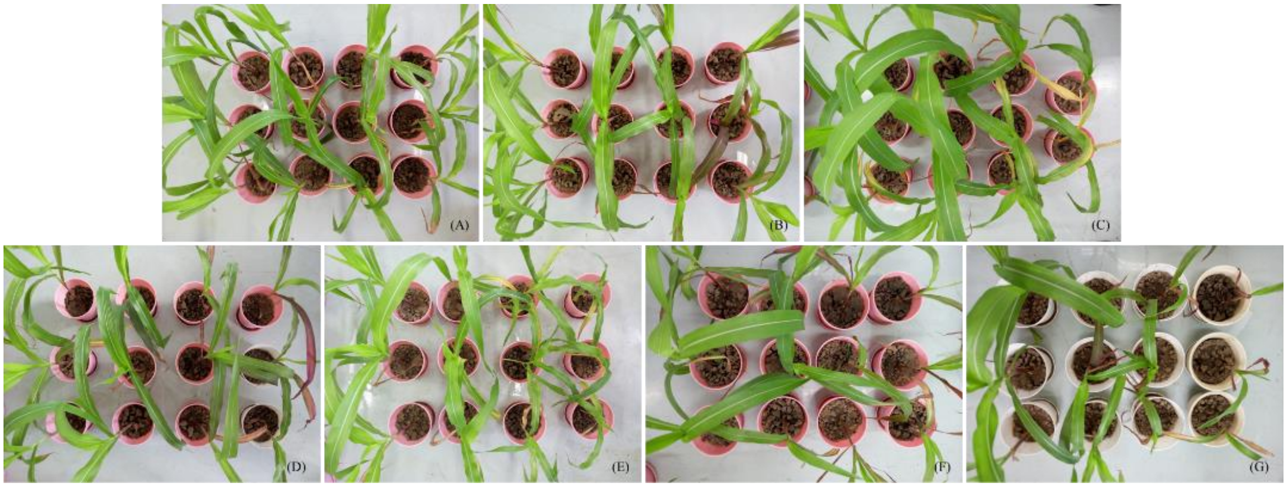

Supplementary Figure 1. Maize plants after 57-day culture under single contaminations of (A) pyrene, (B) Cu, and (C) Cd and joint contaminations of (D) pyrene-Cu, (E) pyrene-Cd, (F) Cu-Cd, and (G) pyrene-Cu-Cd. For each treatment group, the four columns of plants from left to right were exposed to contaminated levels I to IV.

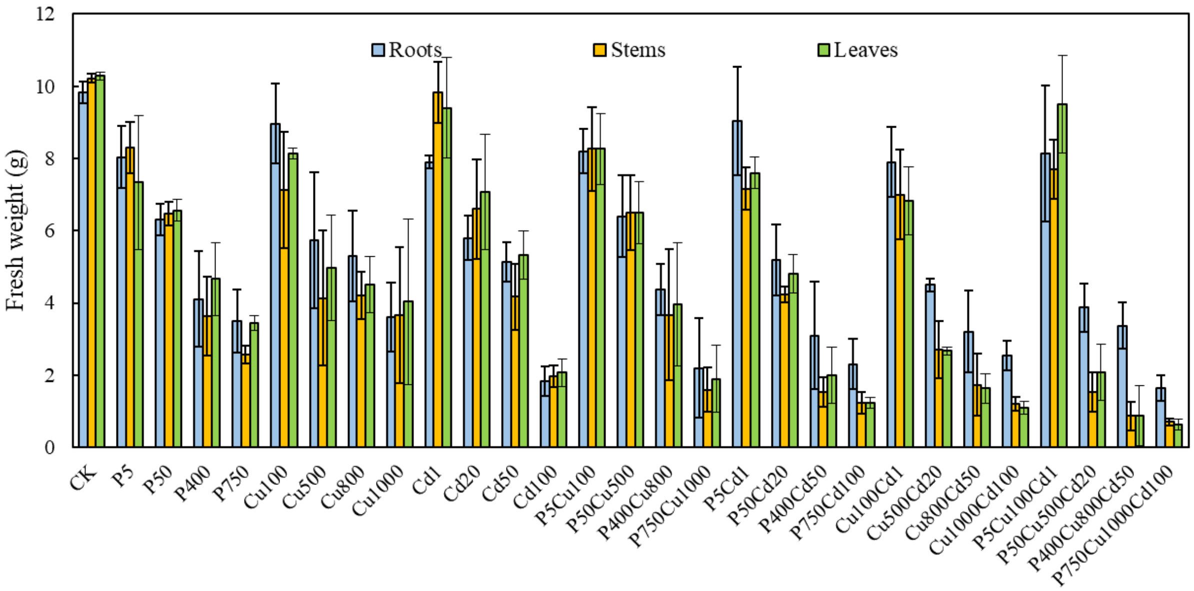

Supplementary Figure 2. Fresh weight of roots, stems, and leaves of maize exposed to varying levels of pyrene, Cu, Cd, and their combinations for 57-day culture. Data are presented as the mean ± SD of triplicates.

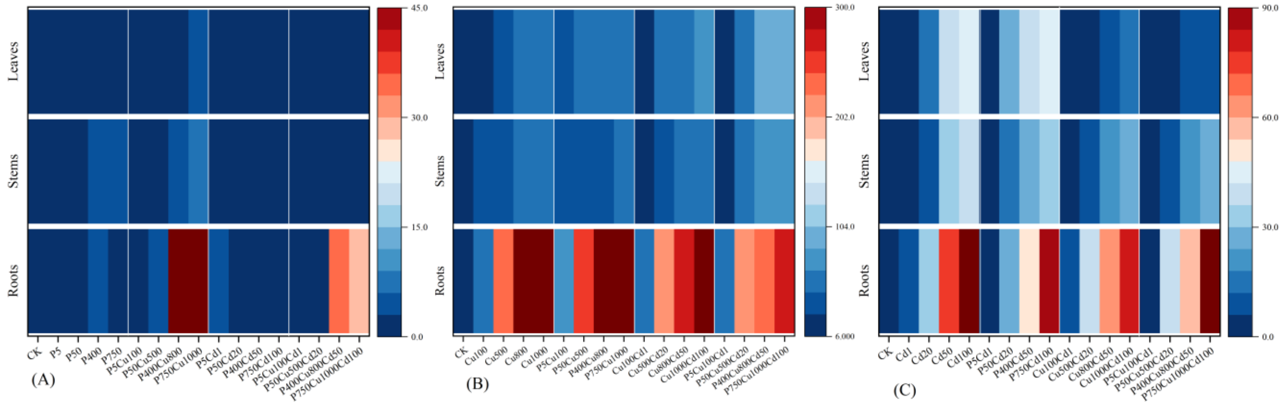

Supplementary Figure 3. Heatmaps of (A) pyrene, (B) Cu, and (C) Cd accumulation in maize roots, stems, and leaves under different treatments. The color bars indicate the concentration in mg/kg.
